# Guidance and surroundings awareness in outdoor handheld augmented reality

**DOI:** 10.1371/journal.pone.0230518

**Published:** 2020-03-19

**Authors:** Aitor Rovira, Ava Fatah gen Schieck, Phil Blume, Simon Julier

**Affiliations:** 1 Dept. of Computer Science, University College London, London, United Kingdom; 2 The Bartlett School of Architecture, University College London, London, United Kingdom; 3 The Regency Town House, Brighton, United Kingdom; University of Milan, ITALY

## Abstract

Handheld and wearable devices are becoming ubiquitous in our lives and augmented reality technology is stepping out of the laboratory environment and becoming ready to be used by anyone with portable devices. The success of augmented reality applications for pedestrians depends on different factors including a reliable guidance system and preventing risks. We show that different guidance systems can be supplementary to provide directions to a point of interest and offer clues that help the user find the augmented data when they get close to the location they have to visit. We tested the helpfulness of a map with the points of interest, an image preview of the next point of interest to visit, and an arrow showing the direction to it. The results show that the effectiveness of these guidance systems depend on the distance to the point of interest and the accuracy of the data obtained from the Global Positioning System. We also measured the total time that participants spent looking at the screen, as well as the perceived elapsed time as a measurement of real world dissociation. Finally, we discuss preliminary results to minimize the risk of accidents when using augmented reality applications in an outdoor urban environment.

## Introduction

Augmented reality (AR) embeds computer-generated multimedia content into the real world [1, 2]. Real world objects can be tracked with a camera and provide the coordinates to overlay virtual objects and visualize them as if they were part of the real environment. Among other applications, AR is becoming popular in outdoor environments thanks to the broad availability of handheld devices. There are several examples of applications that are designed to provide clues about the particular points in space the user needs to look at, for example [3–5]. In this paper, we look at two important factors for the success of this type of applications. First, guidance systems need to provide reliable clues, not only to make people get to the location, but also provide clues about where to look at when near the location. outdoor tracking systems are either not very accurate such as global positioning system (GPS), or require a large amount of data, for example simultaneous localization and mapping (SLAM) techniques. A second factor concerns with safety. Walking about a urban area while keeping the attention to the screen of a handheld device can compromise the user’s safety. An AR experience can be engaging to the extent to make users less aware of the surroundings and not putting enough attention into the events happening around them [6, 7]. They face the paradox between the experience being so engaging that may lead to a higher exposure to a dangerous situation.

The main contribution of this paper is the study of the effectiveness of two different guidance techniques to make people move to specific locations in an outdoor augmented reality application and also providing clues about where they have to look at once they get there. In addition to this, we also measured the time spent looking at the screen can provide an idea about how aware users are to their surroundings. This is an important point that needs to be considered when designing applications to be used in a real world environment such as urban areas to minimize the exposure to dangers.

## Related work

AR offers the possibility to enhance real environments with virtual objects that can assist in different experiences. Examples of this are new ways to visit museums [8–10] and cultural and historical heritage sites [3, 11, 12]. The benefits of using AR are twofold. First, it allows to complement a real world scene by embedding the missing information in it. In the case of historical sites, an AR experience allows to visualize how a place and its inhabitants looked like in the past. Second, navigation systems that overlay signs to provide information on how to get to a specific location, both for indoor environments [13] and outdoors [14, 15].

### Guidance systems

AR has been used extensively to develop navigation systems in order to show directions. Applications designed to use indoors include guides for people through a museum [16, 17] and to optimize the picking order in a warehouse [18]. In outdoor environments, applications face two problems related to navigation. One is the accuracy of the tracking systems used and the other how to guide the user efficiently to each location. Several ways of guidance have been proposed in the past. Some can be subtle, such as a visual saliency system [19] to guide the user’s attention, while others are more popular techniques, such as a map, overlaying direction arrows [13, 20] or showing the user still images and videos enriched with extra objects such as signposts [21].

AR also offers the possibility to show extra content on the locations visited. This information can be overlaid on top of real elements, such as houses, monuments, and landmarks, as if they were part of it. Originally, AR was designed to be used with low-level detailed fiducial markers to provide the coordinates where the augmented data needs to be shown. Current tracking solutions such as ARCore [22], Vuforia [23], and ARKit [24] have tracking capabilities to recognize different 2D and 3D elements of the real environment. A user will need some information about what he is looking for to know where he needs to look at when he gets to the location. Fiducial markers are clearly recognizable [25] and do not present this ambiguity. Nevertheless, it is not always possible to place markers as they can be invasive and could even be removed or concealed easily. Knowing where to look at can be difficult if there are several possible candidates at direct sight [17]. While there has been a lot of research about how to guide a user to a specific location, there are not many solutions about how to provide clues about the viewing direction.

### Safety

AR applications can be very engaging to the extent that can compromise the user’s safety. When the user feels highly dependent on the extra information provided, they might be absorbed to even not putting enough attention to the environment surrounding them. This phenomenon is called cognitive absorption [6] and it is composed of 5 dimensions, including temporal dissociation and attention focus. Also, the concept of absorption in the context of video games has been referred as real world dissociation [26].

Safety in terms of being distracted from the surroundings is a topic that has been barely addressed by the AR community. Some works have mentioned it, but only applied on the field of AR application for cars [1, 20]. Others have mentioned to be a lesser concern in application for pedestrians [27]. However, as we become more dependent on the information provided on electronic devices, people tend to divert the attention to the screen, hence neglecting their own safety in public outdoor environments and the safety of others. This constant provision of information in their devices is what makes people disengage from reality and more exposed to accidents. An AR application can provide a video stream of what it lays ahead and it is arguable that it makes it safer than non-AR applications, the novelty of the experience can make users increase their distraction levels.

Several studies have carried out experiments in the broader area of mobile devices in everyday life and how this affect to our safety. Some articles describe field studies [28–30] that observed the behaviour of people in a crosswalk, studying their responses when they were using portable devices, such as mobile phones and portable music players. Virtual reality has also been used to depict specific dangerous situations in order to study how handheld devices affect their behaviour when they have to cross a street, without exposing pedestrians to the actual danger [31–34].

Different metrics have been used previously to measure pedestrian safety. One is to calculate the difference between the elapsed time and time the participant thought it has passed since the beginning of the route, based on the concept of cognitive absorption [6]. A different approach was to measure their cognitive workload by placing out-of-place objects along the itinerary and ask participants if they recalled seeing any of them [28]. Observational measurements in field studies include counting the number of times other pedestrians were forced to evade a distracted person, studying their behaviour at a crosswalk to evaluate if they stopped, and whether they looked both sides before crossing of they walked straight ahead [28, 29]. The studies that used virtual reality have proposed more complete metrics, including the average start delay after the car passes, the safety time before next car comes, the cross duration, the number of times looking left and right, missing gaps where the pedestrian could have passed between two cars, car hits, and close calls.

## Materials

We developed an application with AR capabilities for handheld devices. The goal was to use it in an outdoor environment to evaluate two important aspects, guidance system and user’s safety. In terms of guidance, we tested whether the information provided allowed the users not only to get to the locations they had to visit but also gave enough information about what they had to point the camera at. In terms of safety, we measured their awareness of their surroundings while using the app.

(**a**) Map with the pins on the locations of the POIs. Red pins mark the ones visited, white pin is the next one to visit, and green the following POIs to visit after next one. Labels on each pin have been added to the image and were not visible in the app.
(**b**) Screen shot of the Camera mode with the arrow and a semi-transparent preview of the image for the next POI.

**Fig 1:**
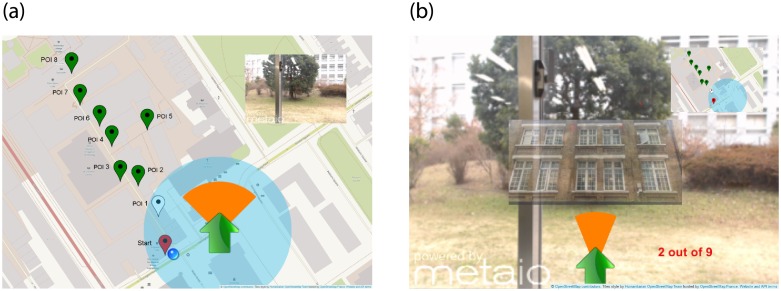
The two modes of the app. The green arrow pointed the straight direction towards the next point of interest to visit. The orange arch indicated the tolerance on the straight direction considering the GPS data accuracy. (a) In Map mode, the app showed the location of the POIs to visit. Tapping on the top right corner changes the view to (b) Camera mode, where the user could see through the camera and depending on the experimental group, the image preview was overlaid.

The application was developed in Unity [35] and had two different modes, Map (Fig 1a) and Camera (Fig 1b). On either mode, the user could tap on the top right corner of the screen to switch to the other mode.

The Map mode was implemented with the use of OpenStreetMap SDK [36] and contained pins on the locations they had to visit. Internally, the app had a list of points of interest (POIs) including the GPS coordinates, the markers, and the augmented data to be displayed on each location. The pins were adjusted manually to make sure their marked the exact location on the map. All POIs were implemented with 2D image-based tracking. The pins on the map showing the locations were coded in different colours, they were either red if the person had already visited the location, white if it was the next location to be visited, and all the locations still to be visited to finish the itinerary were marked in green. The map also showed a blue dot on the current location of the user and a semi-transparent blue circle around to indicate the accuracy range obtained from the GPS data, similar to other popular map applications. The camera mode was implemented using Metaio SDK, which streamed from the integrated camera of the tablet. The same video stream was used for camera tracking on each location. The augmented data shown was not relevant for this study, so we used simple placeholder images to prevent users spending time watching the content.

In this paper, we refer as *target location* or simply *location* a geographical point that is unambiguously represented with GPS coordinates. In our application, the GPS coordinates are 2D vectors, we did not take height into account. A *visual target* is a physical object or part of it of any size situated at a specific location. A *point of interest* (POI) is defined as a location-visual target pair. The *marker* or *target* is the 2D image or 3D object that contains a description of the visual target used by an AR application to detect a POI.

### Guidance systems

In addition of the camera stream and the map, the users also had two additional cues in order to discover the POIs. The first one was an image preview of the next target they had to find, as seen in the centre of Fig 1b. The image was shown when tapping on any pin in the map and could be disabled tapping at it at any time. The semi-transparent image was overlaid on the central part of the screen so it could be used to match it with the video stream. An image preview not only provides graphical information about the next POI, it also helped to estimate the ideal distance and angle from the user to it. The downside of an image preview is that it can be misleading if there are multiple objects that look very similar, such as when looking for a single window on a facade with multiple windows that look very similar or a house on a street with houses of similar shape and colour.

The second guidance system was a widget consisting of an arrow showing the straight direction to the next location and an orange arch, as shown in Fig 1a. The percentage of the circle represented the aperture angle that encompassed the area where the location was, taking into account its estimated position and the accuracy in metres of the data retrieved from the GPS. If the accuracy (in metres) returned a value greater than the estimated distance to the location, then the entire orange circle was painted. That meant the POI was closer than the accuracy range thus it could be in any direction from the current user position.

### Points of interest

**Fig 2:**
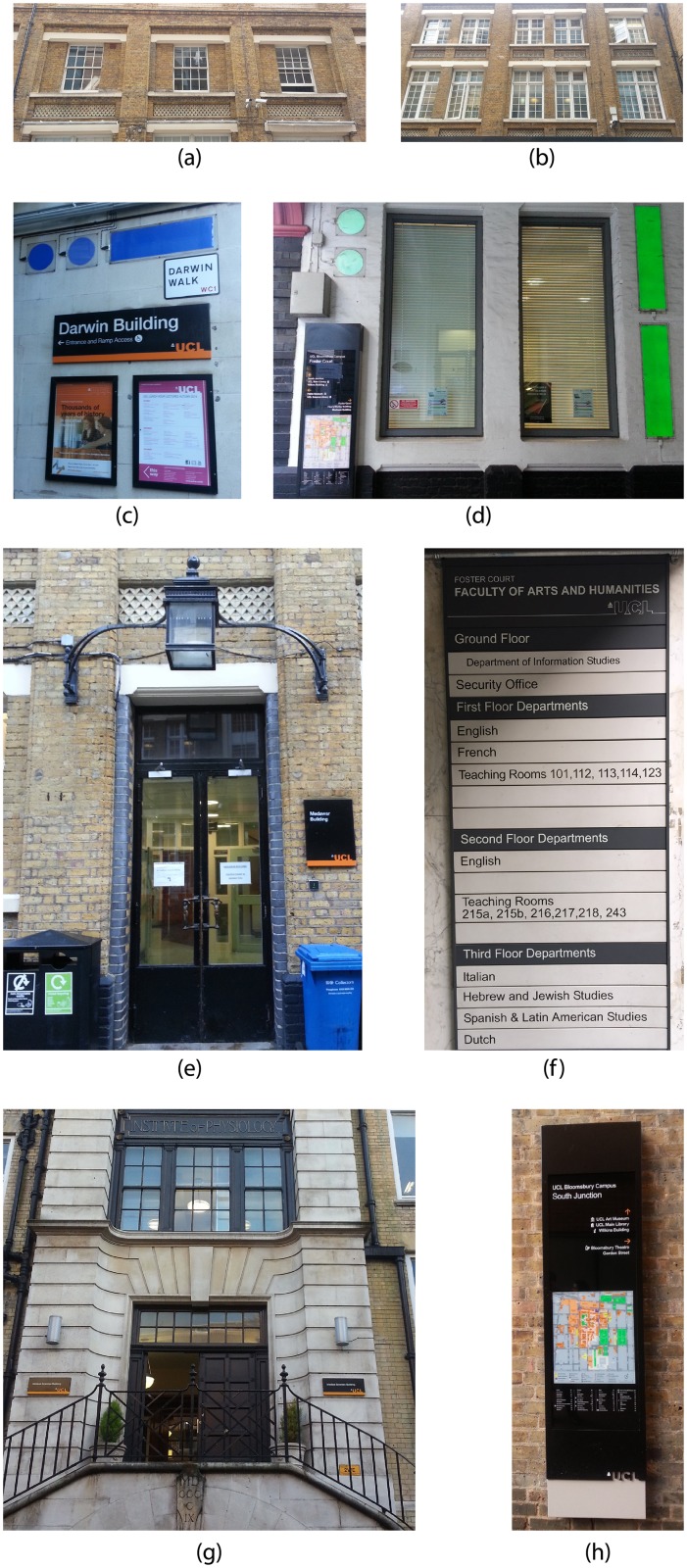
Image for each POI used as markers internally in the AR application. (a) POI 1, (b) POI 2, (c) POI 3, (d) POI 4, (e) POI 5, (f) POI 6, (g) POI 7, (h) POI 8.

The application contained 8 POIs plus a initial one tagged as POI 0, which was used to mark the starting point of the experience. It was designed to visit the POIs in a pre-determined sequence. POI number 1 (Fig 2a) and 2 (Fig 2b) consisted of images of windows on two different facades that contained very similar windows. POI 3 (Fig 2c) and 4 (Fig 2d) were located in two different corners. POI 5 (Fig 2e), participants had to move away from the main alley and go through an area covered by a building. POI 6 (Fig 2f) was a hidden 1-metre high sign next to the door of a building. POI 7 (Fig 2g) was a large section of the facade of a building. POI 8 (Fig 2h) was the same sign used as POI 0 at a different location, also representing the end of the itinerary. The distance from POI 0 to POI 8 was around 200m.

POI 0 (start point) was used to confirm that the app was working correctly. The total time needed to complete the itinerary was calculated from the discovery of POI 0 and finally finding POI 8. We selected these POIs to have a wide selection of visual targets. Some POIs such as POI 3 and 4 were easy to find by just looking at the map, as they were located in corners, while others needed some extra clues. POIs were represented from small elements such as POI 6 and 8 to a big portion of a facade as in POI 7. Some POIs were visually unequivocal such as POI 3, while others were a subset of a repeating pattern, as at POI 1 and 2. Some POIs could be easily found when facing forwards on the walking direction, while in 6 and 7, they had to turn around to find them. At POI 5, the user had to move away from the main alley and go through a pedestrian pass under a building where the GPS signal was lost.

## Methods

Each participant was instructed that they had to walk around an area and find the 8 POIs in the pre-determined sequence. The itinerary was entirely in a pedestrian area to comply with the rules of the UCL Ethics Committee. The map was available for all participants, regardless of the experimental condition. A researcher took them to the starting point and showed how to use the app by pointing the tablet, an iPad mini 3 with 4G connection, to POI 0. The time started counting once POI 0 was discovered and they had to move to POI 1. There was a time limit to find each POI. The time limit was set to 120 seconds for the first three POIs, 90 seconds for the following three and 60 seconds the last two. We initially set the time limit to 60 seconds for all POIs but we decided to allow more time to find the first ones. This decision was made after testing the app with 5 colleagues before the experiment. We found out that users were still trying to familiarise with the app and some missed the initial ones. The researcher instructed them to move to the next one if the time had reached the limit.

The image preview and arrow widget were shown to each participant depending on the experimental condition. The map could be used to estimate the route they would take, and also to get an idea of the direction towards the next location. Participants were instructed about the colour coding of the pins in the map. A POI was found if it was the next one to find on the list and the application could track it once, even if it was for a fraction of a second. Every time a POI was found or timed out, the colour of the pin was updated accordingly.

The goal was to study the total time that each participant would take to visit all POIs and how the two widgets would influence on the total time needed. Our hypotheses were that **H1** Having the clues from either the image preview or the arrow widget available, the total time needed would be reduced compared to the experimental condition where participants only had the map available, **H2** an image preview would reduce the time a participant spent looking at the screen compared to having the arrow widget only or just the map.

### Measuring safety

We aimed to use the concept of absorption [6] and measured the difference between the time needed to complete the itinerary and the time each participant thought it took to complete it. However, this measurement is not very reliable, some people are naturally better than others at estimating the time elapsed and the result would be based on a subjective measure. We also measured the time participants spent looking at the screen. A simple screen saver was implemented and the screen automatically faded out to black after 5 seconds and they had to touch the screen to bring the image back. We instructed them to leave a finger on the screen if they wanted to keep the screen on but we also asked to lift the finger if they were not looking at it. We measured the amount of time the screen was on as an estimation of how much time they spent looking at the screen.

### Post-experience questionnaire

Participants were asked to fill out a questionnaire after the experience to share their thoughts about the app (Table 1). We were interested about their subjective perspective about the information provided and how they used it. The questionnaire was divided in four blocks. The first block referred to the application in general, the last three blocks were about each guidance system tried: the map, the arrow, and the image preview. Each question was answered on a 7-point Likert scale, with the lowest score being 1 when participants completely disagree with the statement, and the maximum score was 7 when they completely agreed with the statement.

**Table 1:** Post-experience questionnaire. The two first blocks were answered by all the participants, while the ones referring to the arrow and the image preview only by those who had each one available.

Code	Question
**About the app in general**:	
AppEasyToUse	It was easy to use.
AppConfusing	Too much information was provided, it was confusing.
AppInfoReliable	The information provided was reliable.
AppInfoSufficient	The information provided was sufficient for me to fulfil my task.
EnhanceExperience	It can enhance the experience of visiting a city.
Awareness	I was aware of what was happening around me while I was using it.
Absorption	I got absorbed while I was using it.
**About the map**:	
MapInfoEasy	The information provided by it was easy to understand.
MapInfoReliable	The information provided was reliable.
MapToLocation	The information provided was useful to get to the location.
MapAtLocation	The information provided was useful to know where to look at once I got to the location.
**About the arrow**:	
ArrowUsed	I used it.
ArrowUnderstand	It was easy to understand how it worked.
ArrowInfoReliable	The information provided was reliable.
ArrowToLocation	The information provided was useful to get to the location.
ArrowAtLocation	The information provided was useful to know where to look at once I got to the location.
**About the image preview**:	
ImageUsed	I used it.
ImageUnderstand	It was easy to understand how it worked.
ImageInfoReliable	The information provided was reliable.
ImageToLocation	The information provided was useful to get to the location.
ImageAtLocation	The information provided was useful to know where to look at once I got to the location.

### Experimental procedures

40 participants (12 females, age range 19-49 years old, age mean±standard deviation = 28±6.3) were recruited around the university campus and via email sent to different departments. They were asked to read the information sheet that briefly described the application and the purpose of the study, and then they were asked to give their written consent in order to participate in the study. A researcher gave them a quick overview of the application in the tablet and then were taken to POI 0 (Start point), where the itinerary started. A few extra clues were given at that point, they were instructed to look for signs, details on the building facades such as a window, and groups of these elements. Targets could be on the ground floor or the first floor level, and that they did not need to go up stairways or enter any building. The researcher then explained how to see the augmented data at POI 0 by pointing the camera towards a sign and revealing on the screen the first image used as augmented data. The researcher then asked them to try it themselves with the same sign. From the moment POI 0 was tracked while the tablet was in participant’s hands, the clock started ticking and no further instructions were given. Furthermore, the researcher walked with them all the time to prevent any potentially dangerous situation and to answer any question that participants could have about how to use the app. They were encouraged to ask questions at any time about it. When they finished the itinerary, they were asked to fill out the questionnaire. It took approximately 30 minutes to complete all the stages of the study and each participant was paid £7 as compensation for the travel expenses and their time.

## Results

There were two binary independent variables that refer whether the image preview (*ImagePreview*) and the arrow widget (*Arrow*) were available, resulting on a 2 × 2 experimental design. 10 participants were allocated to each experimental condition, with no significant differences in age between groups. Participants were assigned to each experimental group alternately as soon as they arrived to the meeting point.

There were two main dependent variables. First, the total time needed to complete the itinerary. In the worst case scenario in which a participant could not find any POI, the time would be the total amount of the time allowed to find each POI, 12 minutes and 30 seconds (750 seconds). The second outcome was the total number of POIs found. Table 2 shows the mean and standard error for the number of POIs found and the time needed to complete the route. At first glance, the number of POIs found looks higher when participants could see the image preview and the results are not improved making the arrow available. Also, these two dependent variables are strongly negative correlated (*r* = −0.84, *P* < 0.0001) as expected, the higher times were obtained when the time had run out.

**Table 2:** Mean and standard errors of the number of POIs found and the time needed to complete the itinerary for each experimental condition.

	Number of POIs found	
	Image Preview	
	On	Off
Arrow		
On	7±0.9	4.0±1.2
Off	7±1.6	3.5±1.0
	Time Elapsed (in seconds)	
	Image Preview	
	On	Off
Arrow		
On	402.5±87.1	552±81.1
Off	423.5±118.2	568±59.0

Looking at the time elapsed, ANOVA confirms that ImagePreview is statistically different and reveals a strong effect size (*F*(1, 36) = 26.904, *p* < 0.0001, partial *η*^2^ = 0.427). On the contrary, Arrow (*F*(1, 36) = 0.326, *p* = 0.572, partial *η*^2^ = 0.009) and the interaction factor Arrow•ImagePreview (*F*(1, 36) = 0.104, *p* = 0.749, partial *η*^2^ = 0.003) are not significant. Shapiro-Wilk test shows that the distribution of the residuals is not ideal, but close to being compatible with normality (*p* = 0.042). An ANOVA on the number of POIs found also shows that ImagePreview is statistically significant (*F*(1, 36) = 74.024, *p* < 0.0001), while Arrow (*F*(1, 36) = 0.651, *p* = 0.425) and Arrow•ImagePreview (*F*(1, 36) = 0.289, *p* = 0.594) are not. Given that the two dependent variables are highly correlated and the ANOVA returns very similar results in both of them, they do not need to be studied separately. We also looked at whether the results could be statistically different based on age (*F*(1, 24) = 0.638, *p* = 0.432) and gender (*F*(1, 24) = 0.469, *p* = 0.5), but neither of the two showed evidence towards that assumption.

Concerning the data from the post-experience questionnaire (Table 1), the question *AppInfoSufficient*, which referred to whether the information provided was sufficient to find the POIs, showed different results when the image preview was available (median±standard deviation, 6.5 ± 1.56) and when it was not available (4.0 ± 1.78, Kruskal-Wallis *χ*^2^ = 15.102, df = 1, p<0.001). Even adjusting *α* with Bonferroni correction (n = 21, *α* = 0.05/21 = 0.002), the difference is still significant. Other general questions related to the app were if it was easy to use (*AppEasyToUse*, median±std.dev. 6±0.99, K-W *χ*^2^ = 0.14, df = 2, p = 0.93), if there was too much information (*AppConfusing*, 1±0.86, K-W *χ*^2^ = 0.004, df = 2, p = 1), if the information provided was reliable (*AppInfoReliable*, 5±1.58, K-W *χ*^2^ = 6.96, df = 2, p = 0.03), and if such an app can enhance the experience of visiting a city (*EnhanceExperience*, 6±1.46, K-W *χ*^2^ = 3.37, df = 2, p = 0.19). The only question that suggests there is a statistical difference between groups is AppInfoReliable. Dunn’s test with Bonferroni correction further reveals that the experimental group that is statistically different from the rest is the one with both the image preview and the arrow available (p = 0.013 at *α*/2 significance level) with higher scores than the rest of groups (median±std.dev., 6±0.82). There were no significant differences among any experimental groups in either question related to the map.

The blocks of questions related to the image preview and the arrow contained the same questions but were filled out only by those who had each either one or both of these guidance systems available. Comparing the results, participants who could see the image preview thought the information was more reliable (*ImageInfoReliable*, median±std.dev., 7 ± 1.37) compared to the feedback provided about the arrow (*ArrowInfoReliable*, 3.5 ± 1.64), more useful to get to the location (*ImageToLocation*, 5.5 ± 1.6, *ArrowToLocation*, 3 ± 1.73), and more useful to know where to look at once the location was reached (*ImageAtLocation*, 7 ± 1.39, *ArrowAtLocation*, 3.5 ± 2.02). We did not add statistical tests to these questions as it was not our goal to compare which one was better between the two.

We look now at safety in terms of the participants’ guess about how much time they took to complete the itinerary. The results show no significant difference among experimental groups, with an overall mean±std. deviation 164.7±167.34s, measured in seconds. The other measure was the amount of time participants spent looking at the screen. The results show again very similar amount of time spent looking at the screen for different experimental conditions, having no significant differences between either Arrow (F(1,36) = 0.523, p = 0.474) or ImagePreview (F(1,36) = 0.308, p = 0.582). The average percentage of time across all groups that participants spent looking at the screen was 86±7.8%. Two questions in the questionnaire were related to surroundings awareness. We asked participants how much they thought about the environment around them (*Awareness*) and their level of absorption while using the application (*Absorption*). Participants reported a high level of absorption with an overall median = 5±1.67 and a statistical tests suggests that there could be significant differences between groups (K-W, median±std.dev. 5±1.67, *χ*^2^ = 130, p = 0.03). Dunn’s test further reveal that participants who could see the arrow reported levels of absorption close to being significant compared to those who did not have it, p = 0.29 for those who could not see the image preview and p = 0.03 for those who had the image available as well, both p values to adjusted to significance level *α*/2.

## Conclusions and discussion

The results confirm H1 partially. Participants who had the image preview available needed less time to find the POIs. The image preview did make it easier to find the POIs, and even participants who had the image available scored higher in the questionnaire when asked about whether the information provided was sufficient compared to those who did not have it, independent from the other experimental variable, Arrow. The number of POIs found was highly correlated to the time needed to complete the itinerary. Participants who found more POIs tended to finish the itinerary faster. The preview images not only showed the building or the detail of the building they had to aim the camera to see the augmented content but also provided information about the ideal angle to look from. On the other hand, we did not find evidence that the arrow helped participants to reduce the time to find them. We speculate that the arrow did not provide reliable data due to low accuracy in relation to the distance between POIs. The arrow can provide useful information in large areas where the user needs to walk long distances between locations, and when the next location is not in direct sight from the current user’s position. In this case, an image preview would not provide enough information and it would be easier to guess the direction compared to the case of having only the locations marked on a map.

Widgets such as the arrow can also provide a more engaging experience but the results suggest there is a chance that participants were more absorbed staring at the screen. About the time participants spent with their eyes on the screen, we cannot conclude that any of the guidance system made the app safer, but participants spent an average of 86% of the time looking at the screen, which is a considerable amount of time. We did not find evidence to support H2. This hypothesis suggested that there could be a reduction of the time spent looking at the screen when the image preview was available. While the arrow needed to be checked constantly to see whether the direction had changed, participants could retain the image in their mind and that would make it not necessary to look at the screen as often as in the case of the arrow. However, the results did not support this idea.

In applications like the one presented in this paper, it is important not only to provide the information needed but also to not provide too much information. Too much information could be confusing and stretch the time needed to learn how it works. The results showed that participants thought there was not enough information when the image preview was not available but there was not too much information in any case.

AR applications can provide engaging experiences by showing extra information. In this paper, we presented an application in a urban area, but AR has also a lot of applications indoors environments, and even in rural areas. However, to have an engaging experience, the application needs to provide enough clues for the user to be able to find the augmented data. This includes both guiding the person to a specific location and providing information about where to look at. In the future, we aim to test these guidance systems in larger areas and try other ways of offering the clues, such as the X-ray vision described by [5]. A map provides an overview of where to go and the route to take to get there, an arrow shows what direction to take and also can make it easier to find the POI when it is nearby. However, GPS data does not provide the accuracy needed when getting in the range of a few metres. In the last stage, an image preview of the target can help but only when there is a visual target. In cases where there are not landmarks e.g. a house that was demolished, it is obvious that an image would not provide any help and be more distracting than helpful.

We wanted to add a short discussion about the long list of extraneous variables that could have an impact of the results. The role of previous experience with similar apps, the proficiency on reading maps, or simply the state of mind at the time when a participant tested our app could have a strong effect. We tried to minimize the effect caused by variations among individuals by randomizing participants. We also tested if there was any effect caused by the age and gender of the participants, but we did not find evidence on that direction. Other variables external to the participant can also change the results. One of them is how busy the street is at the time of the experience. For this, we avoided carrying out the study during the busiest hours of the day, when simply the number of pedestrians in the area can slow down participants while completing the itinerary. We also tried to avoid scheduling participants during the early hours in the afternoon on sunny days because some POIs were more difficult to track by the app due to the reflection of the sunlight on the glass windows. This case was especially for POI 1 and POI 2, both were on facades facing southwest. In summary, there are multiple variables that can change the results and it is not feasible in a study of this size to control all of them. However, we tried to minimize their effect when we had alternatives to do so.

Safety is a subject that has been studied in AR heads-up displays cars superimposing relevant information on the windshield of the vehicle, but it is a concept that should not be neglected when designing applications for pedestrians. This is extremely important when using handheld devices, as the user diverts their attention to a small screen on their hands. It has been observed that pedestrians can be less aware of the events happening around him when listening to music or using the phone [28, 29]. In AR applications, this can be especially problematic if users keep looking at the screen of handheld devices. Their attention is diverted from potential dangers that are present in urban areas, similar to the loss of attention of pedestrians while typing on a mobile phone. AR can provide an engaging experience and they might seem safer as the camera provides constant streaming on the display, but trying to find a POI or watching the content require a significant amount of cognitive workload, making users more absorbed on thus neglecting their own safety.

In order to avoid dangerous situations that can put users’ safety at risk, AR applications need to be designed taking them into account without hindering the experience that it provides. For example, one idea would be to add restrictions and show the augmented data only from specific spots to ensure that the users stay away from the street car lanes. We saw during initial piloting that AR is a technology that many people are not familiar with, and made them more absorbed trying to understand how it worked. A few times we had to warn them that they were about to bump into another pedestrian. Another safe alternative is to provide audible clues so the user does not need to look at a screen.

## Supporting information

S1 Data (XLSX)

S2 Data (7Z)
